# Screening of *Candida albicans* GRACE library revealed a unique pattern of biofilm formation under repression of the essential gene *ILS1*

**DOI:** 10.1038/s41598-019-45624-y

**Published:** 2019-06-24

**Authors:** Anna Carolina Borges Pereira Costa, Raha Parvizi Omran, Tuana Oliveira Correia-Mesquita, Vanessa Dumeaux, Malcolm Whiteway

**Affiliations:** 1 Department of Biology, Concordia University, Montreal, Canada; 2 Department of Biology, McGill University, Montreal, Canada; 3 PERFORM Centre, Concordia University, Montreal, Canada

**Keywords:** Biofilms, Fungal genomics, Fungal genes

## Abstract

*Candida albicans* biofilm formation is governed by a regulatory circuit comprising nine transcription factors which control a network of target genes. However, there are still unknown genes contributing to biofilm features. Thus, the GRACE library was screened to identify genes involved in mature biofilm development. Twenty-nine conditional mutants were selected for a second screening revealing three groups of genes: twenty- two conditional mutants were defective for normal growth and unable to form biofilms; six strains, conditionally defective in genes *ARC40, ARC35, ORF19.2438, SKP1, ERG6*, and *ADE5,7* that are likely essential or involved in general cell processes, grew normally as free-floating cells but produced less biofilm; finally, the conditional strain for a putative essential isoleucyl- tRNA synthetase gene, *ILS1*, was unable to grow as yeast-phase cells but was capable of producing a tridimensional biofilm structure in spite of reduced metabolic activity. This unique biofilm still relied on the classical biofilm genes, while it differentially induced groups of genes involved in adhesion, protein synthesis, cell wall organization, and protein folding. Although the conditional mutant repressed genes annotated for morphology and homeostasis processes affecting morphology and metabolism, the dynamic cell growth enabled the formation of a complex biofilm community independent of *ILS1*.

## Introduction

*Candida albicans* is a commensal fungus and a common component of the human microbiota of mucosal surfaces. It can cause both superficial yeast infections like thrush and vaginitis, as well as deep-seated systemic infections that can be life-threatening for immunocompromised individuals^1^. On the host surface or on medical devices such as catheters and prostheses, *C. albicans* can form a monospecies biofilm composed of yeast, pseudohyphae, and hyphal cells embedded in a self-produced extracellular matrix consisting of proteins, carbohydrates, lipids, and nucleic acids^2,3^. These sessile communities start with colonization of a substratum by adherence of yeast cells. Subsequently, the community grows and produces pseudohyphal and hyphal cells. The three-dimensional architecture is formed by these three cell types and the accumulation of an extracellular matrix. In the mature stage, the biofilm releases dispersal cells, mainly budding cells, which can colonize distant sites and initiate new communities^3,4^. The biofilm cells are highly resistant to immune cells and to antifungal drugs such as azoles, and are only sensitive to high concentrations of amphotericin B and echinocandins; therefore, they currently represent a major medical challenge^5–7^.

The molecular mechanism that governs *C. albicans* biofilm formation has been characterized in part based on our knowledge about the transcription factors that control biofilm development^8,9^. A regulatory transcriptional network involves six transcription factors (Efg1, Tec1, Bcr1, Ndt80, Brg1, and Rob1) that positively regulate each other’s expression and globally control the expression of about 1,000 target genes^8^. These target genes are implicated in important characteristics of biofilm formation such as hyphal formation, adhesion, drug resistance, and the production of extracellular matrix as well as processes unrelated to biofilms^3,8,10^. The six transcriptional regulators are required for both *in vitro* and *in vivo* biofilm growth and can activate or repress their target genes directly^8^. The core biofilm circuit has been expanded to include three other transcription factors, Rfx2, Gal4 and Flo8, with specific roles for biofilm formation. Flo8 is required for all stages of biofilm formation while Rfx2 and Gal4 are negative regulators of intermediate time points^9^. This complex regulatory circuitry enables *Candida* cells to sense and respond to multiple environmental stimuli^10^. Besides the master transcriptional regulators, the literature has identified 41 additional transcriptional factors and pointed out 101 non-regulatory genes with established roles in biofilm formation^3^. Although we know the elements that regulate and control *C. albicans* biofilm development, many of the target genes of the biofilm network have not yet been characterized.

Many studies in the literature have monitored the transcription profiles of biofilm cells compared to their planktonic counterparts under different conditions and timepoints to generate valuable insights into how *C. albicans* biofilms are built^8,9,11–15^. These studies identified biofilm-specific sets of genes that inspired other groups to construct null mutants and search for their biofilm function^16,17^. However, the expression level of a gene is not predictive of a given phenotype for a strain possessing mutations in the same environmental condition^18^.

In this study, the GRACE (gene replacement and conditional expression) collection was screened for biofilm formation. This mutant library contains 2358 heterozygous mutant strains of *C. albicans* in which the expression of the remaining wild type allele of a gene is governed by a tetracycline-repressible promoter^19^. The biofilms were grown in RPMI-MOPS medium in order to identify genes involved in biofilm development under a strong biofilm-inducing condition. The screening revealed that 29 conditional mutants produced altered biofilms under these conditions. The 29 selected strains were screened again for other biofilm features and for planktonic growth kinetics. The second screening revealed 22 conditional mutants that were defective for normal growth in liquid media and for biofilm growth indicating that a generic growth defect could be responsible for the abrogated biofilm phenotype. Six conditional mutants for putative essential genes or genes involved in general processes were able to grow in planktonic condition but failed to form a mature biofilm. Surprisingly, the conditional mutant for the putative essential isoleucyl-tRNA synthetase *ILS1* did not grow in planktonic conditions but formed a biofilm comparable to that of the heterozygous and the wild-type strains although the metabolic activity was lower. This *ILS1*-independent biofilm was investigated by the analysis of its transcription profile; this showed that while *ILS1* was strongly shut off, three other amino-acyl tRNA- synthetases were upregulated relative to their normal expression in the wild-type biofilm, and alternative biofilm genes were modulated to allow the growth of a sessile community in the absence of the essential tRNA synthetase.

## Results

### Biofilm screening of GRACE library

**Figure 1:**
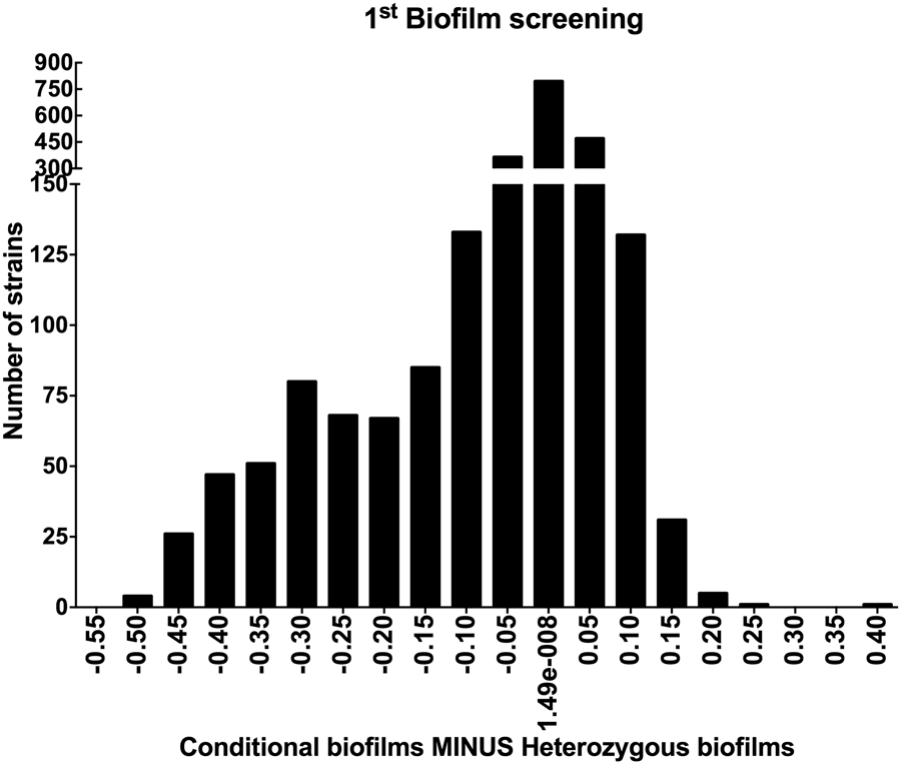
The first biofilm screening revealed that most of the altered phenotypes created defective biofilms. The data displays OD_600_ measurement for the difference between the averages of biofilm growth for each conditional mutant minus heterozygous mutant values. The biofilms were grown in RPMI-MOPS at 37 °C for 48 h. The experiment was performed in duplicate on two different occasions.

In the first screening, 2358 GRACE strains were tested for biofilm formation. Initially, 430 genes identified as essential in the gene essentiality test on solid medium were ruled out of the analysis (Supp. 1). Then, the conditional mutants that showed altered biofilm phenotypes (OD_600_) were identified. The selection focused on the best hints in the first 10% of results for the conditional mutants which formed more extensive or less extensive biofilms (Conditional biofilms MINUS Heterozygous biofilms) coupled with scanning by eye (Fig. 1 and Supp. 1). The screening showed that the suppression of the genes was more likely to repress biofilm development than to increase biofilm growth (Fig. 1). Based on the results, we selected 29 GRACE strains for a second biofilm screening using more controlled biofilm growth conditions and more quantitative analyses. These strains were also examined for planktonic growth kinetics.

### Second biofilm screening for the selected conditional mutants

**Figure 2:**
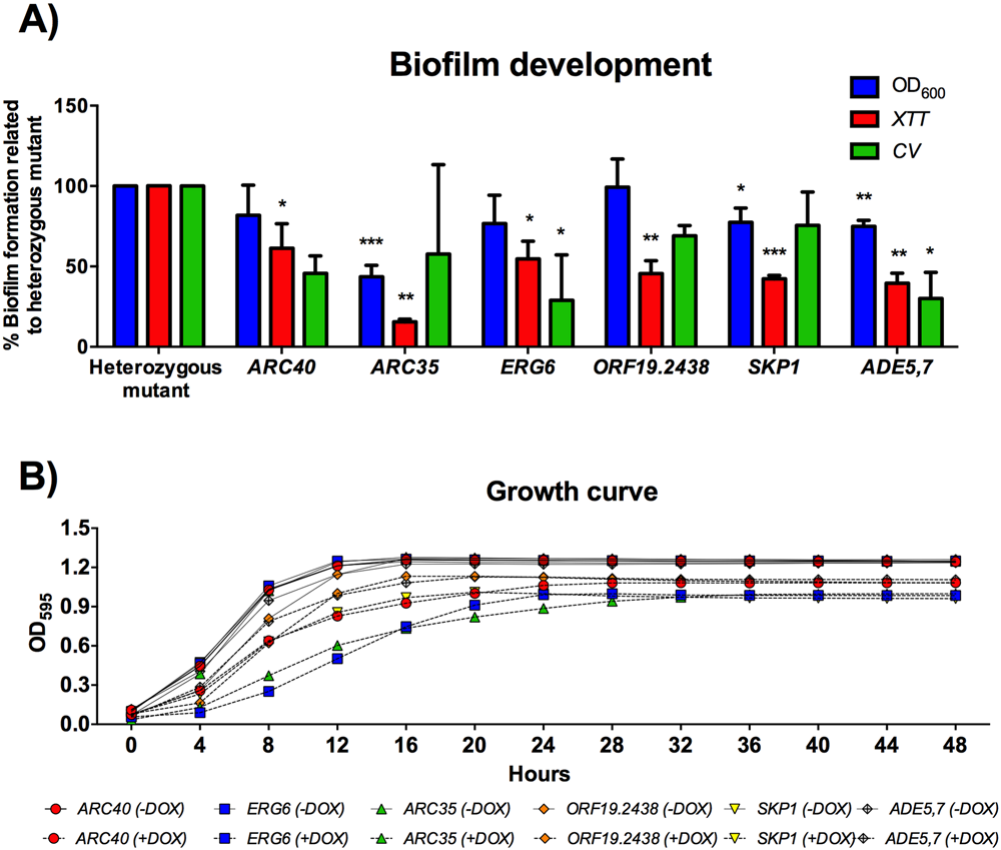
The second round of screening identified a group of genes involved in general processes capable of growing *in vitro* but deficient for biofilm formation. (**A**) Percentage of biofilm formation for the conditional mutant related to the heterozygous mutant (non-DOX) for OD_600_ measurement, metabolic activity (XTT assay), and biomass accumulation (CV assay). The biofilms were grown in RPMI-MOPS at 37 °C for 48 h. (**B**) Planktonic growth curve for the strains grown in YPD −/+ DOX at 30 °C for 48 h. The experiments were performed in triplicate on three different occasions. *t* test, *P < 0.05, **P < 0.01, ***P < 0.001.

In the more detailed second screening, OD_600_ measurement, XTT assay, crystal violet assay, and planktonic growth curve, 22 of the conditional mutants were found to be defective in growth under standard conditions of temperature and growth medium (YPD at 30 °C/48 h). This suggests that this set of genes are directly required for proliferation instead of having specific functions for biofilm development (Supp. 2 Figs 1, 2). The second round of screening also identified 6 conditional mutants that had relatively normal growth kinetics in the presence of doxycycline, but presented altered phenotypes for biofilm formation with statistically significant differences in more than one type of analysis (Fig. 2). The predicted function description for each gene and biofilm findings are summarized in Supplemental Table 1.

### Biofilm formation and cell morphology for the *ILS1* conditional mutant

**Figure 3:**
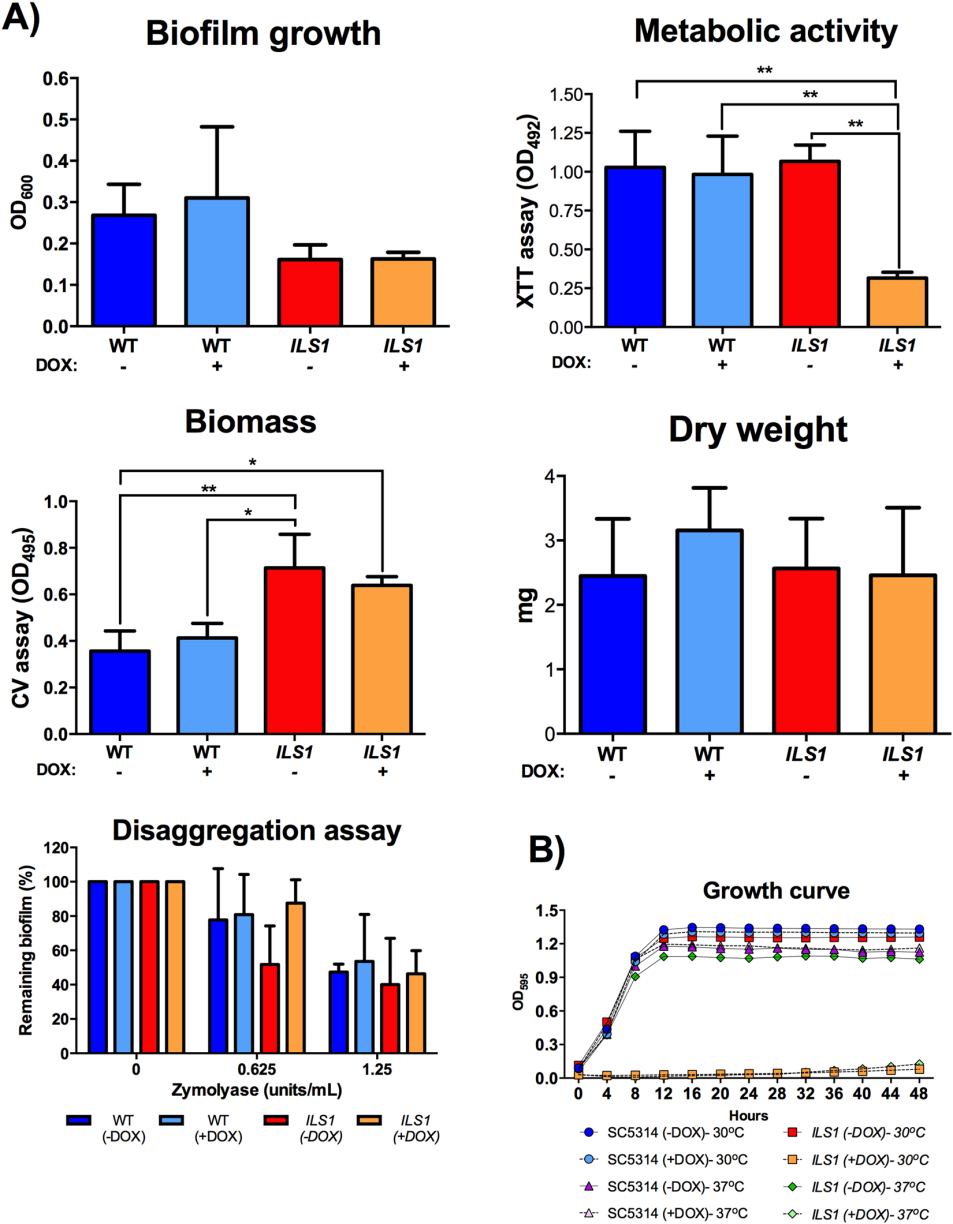
The *ILS1* conditional mutant formed comparable biofilms though it was not able to grow *in vitro*. (**A**) The wild-type (WT) and the *ILS1* GRACE strain biofilms were assessed as biofilm growth (OD_600_), metabolic activity (XTT assay), biomass accumulation (CV assay), dry weight, and biofilm disaggregation assay. The biofilms were grown in RPMI-MOPS at 37 °C for 24–48 h −/+ DOX. (**B**) Planktonic growth curves for the strains grown in YPD −/+ DOX at 30 °C and 37 °C were monitored for 48 hours. The experiments were performed in triplicate on three different occasions. Tukey’s test, *P < 0.05, **P < 0.01.

We observed that while the conditional mutant for the gene *ILS1* failed to grow in planktonic conditions in YPD, the strain readily formed biofilms when compared to both the heterozygous mutant and the wild- type strains (Fig. 3). This gene encodes a putative isoleucyl-tRNA synthetase, with an orthologous gene in *Saccharomyces cerevisiae* also called *ILS1*^20^. In both organisms, *ILS1* null mutants are inviable under standard growth conditions^20,21^. The *C. albicans ILS1* conditional mutant was incapable of growth in liquid YPD at either 30 °C or 37 °C during 48 hours of incubation (Fig. 3B). YPD is the medium of choice to grow free-floating cells in the yeast form needed to initiate biofilm growth^22^. However, the conditionally repressed strain formed biofilms comparable to the wild- type and the heterozygous mutant strains in relation to growth, dry weight measurements and biomass accumulation (Fig. 3A), while the metabolic activity of the conditional mutant biofilm cells was reduced relative to the wild-type (WT) and the non-repressed strains (Fig. 3A).

The biofilm structure formed by the *ILS1* conditional mutant was comparable to the biofilms formed by the heterozygous mutant and by the wild- type strains but had a lower metabolic activity. This might indicate that while the *ILS1* biofilm is composed of fewer or metabolically compromised cells, the conditional biofilm formed comparable biofilm mass, as determined in biofilm growth, dry weight, and biomass accumulation analysis, due to higher production of extracellular matrix. Therefore, we investigated whether the *ILS1*-repressed biofilm produced more extracellular matrix. Twenty-four-hour biofilms were treated with two different concentrations of the enzyme zymolyase which has 1,3 β-glucanase, mannanase, protease, and endochitinase activities^23^, to initiate biofilm disaggregation. There were no statistical differences for biofilm disaggregation for any concentration among the tested strains, suggesting that *ILS1* conditional biofilm phenotype was not due to enhanced extracellular matrix formation, and might be due to altered cell morphology or biofilm architecture (Fig. 3A).

**Figure 4:**
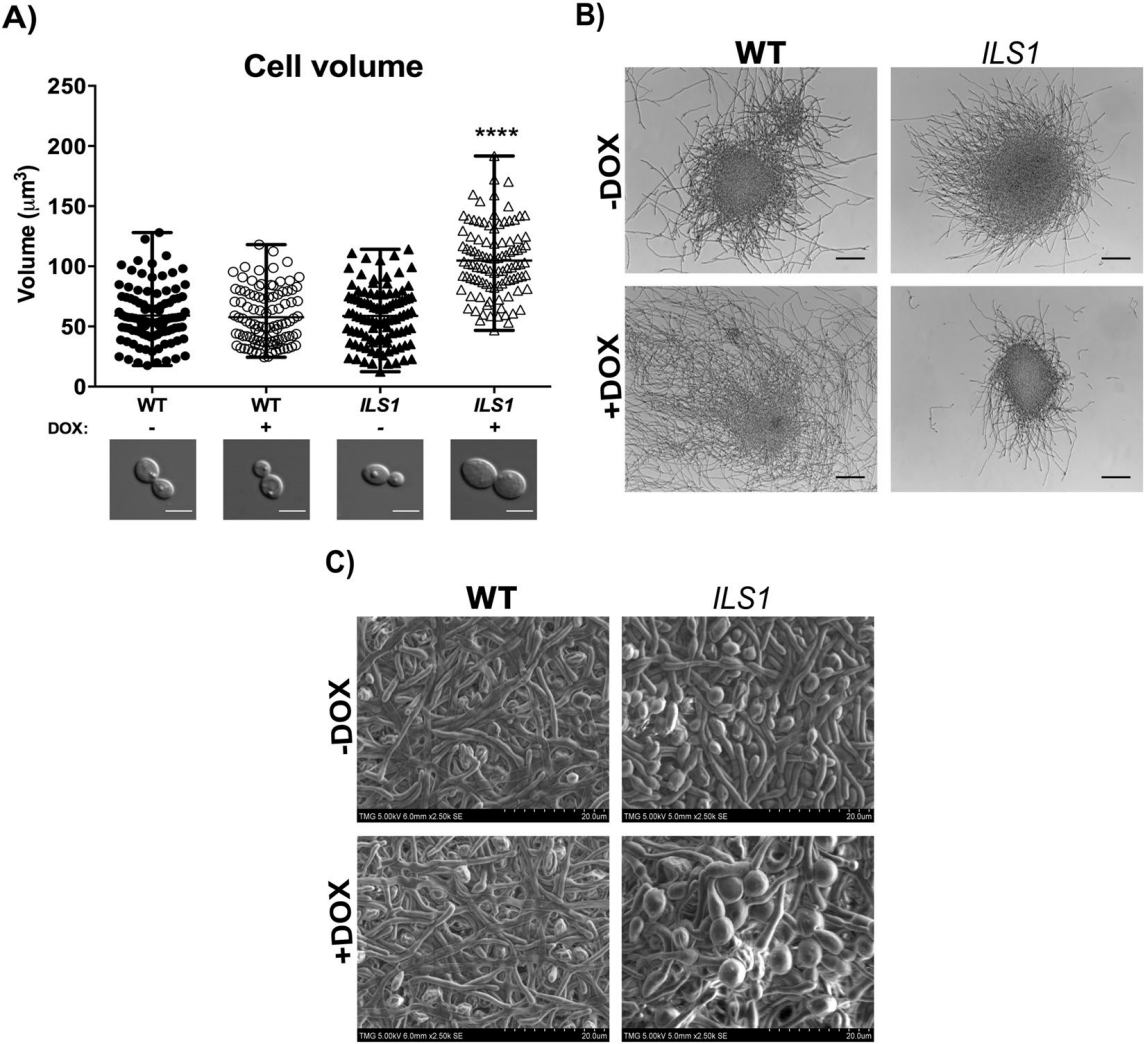
The *ILS1* conditional mutant cells presented altered morphologies in either planktonic yeast and hyphal growth or biofilm phase growth. (**A**) The WT and the *ILS1* GRACE strains were grown in YPD (−/+ DOX) at 30 °C/overnight and imaged to determine the cell morphology and volume. Each column shows 100 cells from three independent experiments, Tukey’s test, ****P < 0.0001. DIC representative images for each strain, scale bars represent 5 µm. B) The WT and the *ILS1* GRACE strains were grown in RPMI-MOPS medium (−/+ DOX) at 37 °C/overnight/220 rpm and imaged to determine the cell morphology. DIC representative images for each strain, scale bars represent 100 µm. C) Scanning electron microscope images for the WT and the *ILS1* GRACE strains growing biofilms on silicon squares pre-treated with bovine serum and grown in RPMI-MOPS medium (−/+ DOX) at 37 °C/48 h, 2500x magnification.

To investigate cell shape and biofilm organization, planktonic yeast cells grown in YPD −/+ DOX at 30 °C/overnight were imaged to determine the cell volume. The WT strain contained budding cells with cell volume averages of 59.06 and 57.65 µm^3^ −/+ DOX, respectively. The *ILS1* heterozygous mutant (-DOX) also presented budding cells with cell volume average of 58.70 µm^3^ (similar to the wild-type cells −/+ DOX) (Fig. 4A). In contrast, the rare detected *ILS1* conditional mutant cells were considerably enlarged (104.71 µm^3^) and rounded, and contained daughter cells as big as the mother cells. This size difference was highly statistically significant (*P* < 0.0001) when compared to the wild-type strain (−/+ DOX) and the untreated heterozygous mutant (Fig. 4A). The planktonic hyphal growth was investigated in RPMI-MOPS and Spider medium. In the RPMI-MOPS medium, both WT (−/+DOX) and heterozygous mutant strains formed microcolonies of hyphae and long branched hyphae while the *ILS1* conditional mutant grew a few short hyphae and small microcolonies of hyphae (Fig. 4B). On the order hand, in Spider medium the *ILS1* conditional mutant formed a few long branched hyphae whereas the WT (−/+DOX) and the heterozygous mutant strains produced a few longer hyphae and many budding cells (Supp. 2 Fig. 3). Although the Spider medium is considered a hyphal-inducing condition, the planktonic mode of growth (vigorous shaking, 220 rpm, and growth in conical tubes) may have interfered in the proper filamentation by the WT and the heterozygous mutant strains different from the *ILS1* conditional mutant which thrived in this condition by producing only filamentous cells.

In the biofilm phase growth, both the wild-type (−/+ DOX) and the *ILS1* heterozygous mutant strains formed mature and compact biofilms composed of many layers of intertwined hyphae, pseudohyphae and yeast cells with channels among the cells (Fig. 4C). Although unable to grow planktonically in yeast form, the *ILS1* conditional mutant was able to produce a mature biofilm composed of layers of different fungal morphotypes, but these presented a distinct morphology. The *ILS1* conditional mutant biofilm was composed of defective and distorted fungal structures showing enlarged filaments with different thickness along the fungal structure. The yeast cells also showed enlarged and globular shapes which were similar to the shapes observed for the rare planktonic cells. There were also enlarged germ tubes and pseudohyphae that seemed to be dividing by stretching the cell (Fig. 4C). Although presenting abnormal biofilm cells, the *ILS1* biofilm architecture provided a similar biofilm mass to that of the heterozygous and the wild-type strains.

### Biofilm transcriptional analysis

The ability of *C. albican*s to construct a biofilm in the absence of the essential gene *ILS1* raises the questions of whether the tetracycline-repressible promoter regulating *ILS1* was properly shut off in the sessile community, whether other tRNA synthetases were up-regulated in a non-specific way to potentially compensate for loss of *ILS1*, and what “building blocks” were involved in the construction of this *ILS1*-independent biofilm. To gain insight into these questions, the *ILS1* conditional biofilm was analyzed at the transcription level. We initially established the transcription expression profiles for the comparison of wild-type cells forming a mature biofilm in RPMI medium (WT biofilm) versus cells grown planktonically (WT planktonic cells). In this biofilm model close to 1500 genes were up-regulated (>1.5 fold-change) and 1000 were down-regulated (<−1.5 fold-change) relative to the planktonic cells (Supp. 3).

**Figure 5:**
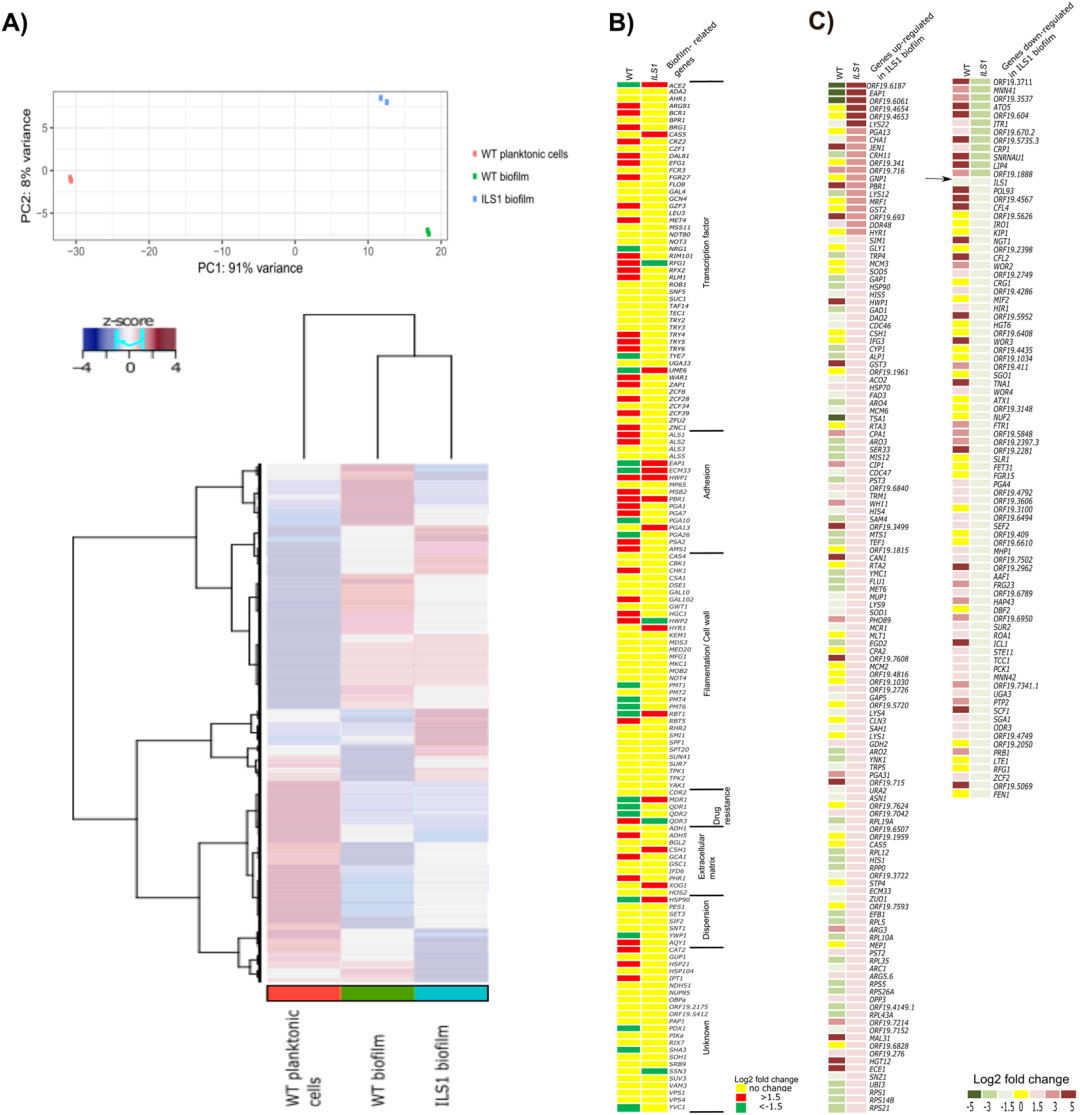
The *ILS1* conditional mutant constructed a unique biofilm regulating the classical biofilm-related genes and key elements. (**A**) Principal component analysis (PCA) and heatmap of RNA-seq data of the WT planktonic cells, the WT biofilm, and the *ILS1* biofilm. (**B**) Heatmap of differential expression of biofilm-related genes^3^ in the RPMI biofilm (WT biofilm vs. WT planktonic cells) and the *ILS1* conditional biofilm (*ILS1* biofilm vs. WT biofilm). (**C**) Heatmap of up-regulated (>1.5 fold- change, adjusted p-value < 0.01) and down-regulated (<−1.5 fold- change, adjusted p-value < 0.01) genes in the *ILS1* conditional biofilm compared with the WT biofilm (RPMI biofilm). The single down-regulated gene in both biofilms, *ILS1*, is pointed out by the arrow.

The principal component analysis (PCA) of the RNA-seq data obtained for the WT planktonic cells, the WT biofilm, and the *ILS1* biofilm revealed that 91% of the variance in gene expression was captured by principal component (PC) 1 which clearly separated the WT planktonic cells from both biofilms, while only 8% of variability captured by PC2 separated the WT biofilm from the *ILS1* biofilm (Fig. 5A). Indeed, the overall transcription profiles of both biofilms (*ILS1* and WT) were more similar to each other than the WT planktonic and biofilm profiles (Fig. 5A).

To generate the *ILS1* conditional biofilm expression profile, the *ILS1* biofilm RNA-seq data was compared with the wild-type biofilm (RPMI biofilm) data since the *ILS1* planktonic cells were inviable. Figure 5B displays the expression in RPMI biofilm and the *ILS1* conditional biofilm of a list of 144 established biofilm-related genes^3^. The RPMI biofilm (WT biofilm vs WT planktonic cells) up-regulated 43 biofilm genes and down-regulated 20 genes. The transcription expression profiling of the *ILS1* biofilm (*ILS1* biofilm vs. WT biofilm) identified 14 up and 4 down-regulated members of this group of biofilm-related genes (Fig. 5B). The up-regulated genes in the *ILS1* biofilm for the groups of transcription factors (*ACE2*, *CAS5*, and *UME6*), adhesion (*EAP1, ECM33*, and *PGA13*), filamentation/cell wall (*HYR1* and *RBT1*), extracellular matrix (*CSH1* and *XOG1*), drug resistance (*MDR1*) and dispersion (*HSP90*) were not differentially expressed in the RPMI biofilm (Fig. 5B). Three out of four down-regulated genes in the *ILS1* biofilm were in fact up-regulated in the RPMI biofilm (*RFG1*, *HWP2*, and *QDR3*) (Fig. 5B). The biofilm adhesion genes *HWP1* and *PBR1* were up-regulated in both biofilms (Fig. 5B).

The conditional repression of *ILS1* in the biofilm did influence the expression of a number of genes relative to the WT cells. The *ILS1*-repressed biofilms significantly induced (>1.5 fold change, adjusted p-value < 0.01) the expression of 133 genes and reduced (<−1.5 fold change, adjusted p-value < 0.01) the expression of 86 genes (Fig. 5C). In the set of down-regulated genes, *ILS1* was among the top 15 most repressed genes (−2.7-fold change, adjusted p-value < 0.0001) indicating that the addition of 20 mg L^−1^ doxycycline was sufficient to shut off the gene in the biofilm phase growth (Fig. 5C). To overcome the lack of this essential amino-acyl tRNA-synthetase, we investigated whether other amino-acyl tRNA synthetases were up-regulated to potentially enable biofilm growth through some generic replacement of the missing activity. While thirty-five out of 36 *C. albicans* amino-acyl tRNA-synthetase transcripts were repressed in the wild-type biofilm, three (arginine- tRNA (*ORF19.3341*), glutamine- tRNA (*GUS1*), and phenylalanine- tRNA (*FRS2*)) synthetases were significantly de-repressed in the *ILS1* biofilm (1.1, 1.5, and 1.2-fold up-regulated, respectively, adjusted p-value < 0.0001, See Supp. 3). These three amino-acyl tRNA synthetases could be candidates to allow some bypass the missing *ILS1* function.

The comparison of the WT biofilm expression profiles with either the *ILS1* biofilm and the WT planktonic cells profiles highlight some interesting differences (Fig. 5C). Among the 133 genes overexpressed in ILS1 biofilm compared with the WT biofilm, only 29 genes were overexpressed in the WT biofilm compared with the WT planktonic cells while 71 of these genes were underexpressed in the WT biofilm compared with the WT planktonic cells. For the group of underexpressed genes in the *ILS1* biofilm, only one gene, the conditionally shut off *ILS1* gene was also observed in the WT biofilm compared with the WT planktonic cells, and 60 out of 86 underexpressed genes in the *ILS1* biofilm were in fact overexpressed in the wild-type biofilm compared with the WT planktonic cells (Fig. 5C).

The *ILS1* biofilm up-regulated a set of unique GPI-anchored cell wall proteins (*EAP1, ORF19.4653, PGA13, CRH11, HYR1*, and *ECM33*) and an adhesion-like protein (*SIM1*) known to be involved in biofilm formation that were either down-regulated or not changed in the WT biofilm (Fig. 5C). At the same time, both the wild-type and the *ILS1* biofilms up-regulated other genes associated with biofilm formation, such as the genes for the adhesins *HWP1* and *PGA31*, the RPMI biofilm induced *WH11*, *DPP3* required for farnesol biosynthesis, the candidalysin *ECE1*, and uncharacterized genes induced in biofilms (*ORF19.716, ORF19.693, ORF19.6840, ORF19.3499, ORF19.7608, ORF19.2726, ORF19.715, ORF19.7042*, and *ORF19.276*) (Fig. 5C).

The categorisation of the up-regulated and down-regulated genes in the *ILS1* biofilm compared with the WT biofilm revealed most of the differentially expressed genes were involved in response to stress, regulation of biological process, transport, response to chemicals, interspecies interaction between organisms, and organelle organization (Supp. 2 Fig. 4). The up-regulated genes were enriched for GO terms for translation, ribosome biogenesis, cell wall organization, RNA metabolic process, and protein folding (Supp. 2 Fig. 4). Notably, the *ILS1* biofilm up-regulated the molecular chaperones *HSP90* and *HSP70* at levels at least 2.4- fold higher than the wild-type biofilm, though *HSP70* was already up-regulated in the wild-type biofilm relative to the planktonic state. There was a high number of down-regulated genes in the *ILS1* biofilm in processes critical for morphology, such as filamentous growth, cytoskeleton organization, pseudohyphal growth, and growth of unicellular organism as a thread of attached cells, which may explain why the *ILS1* biofilm cells appear distorted and defective (Fig. 4C). Furthermore, there was a large number of genes annotated in the cellular homeostasis processes involved in cooper (*CRP1* and *ATX1*) and iron metabolism (*IRO1, CFL2, FTR1*, and *HAP43*) that were down-regulated in the *ILS1* biofilm compared with the WT biofilm while the same genes (*CRP1, CFL2, FTR1*, and *HAP43*) were up-regulated in the WT biofilm compared with the WT planktonic cells (Fig. 5C).

## Discussion

Approximately one-third of the *C. albicans* genome was submitted to a large-scale genetic analysis of biofilm formation. This screening of 2358 GRACE heterozygous mutant strains treated or non-treated with doxycycline identified several genes whose conditional inactivation impacted biofilm formation (29 genes) and suggested that these genes were more likely involved positively in biofilm formation than negatively. Biofilm suppressor genes might have been under-represented in our study because the medium chosen to promote biofilm growth in this study supports the formation of robust biofilms which can reach a saturation threshold and hide overgrowth phenotypes^24,25^.

Although 430 candidate essential genes on solid media were ruled out before the selection of the hints, 22 conditional mutants selected in the first screening still showed severe defects for general growth in liquid media which could explain the poor biofilm growth. O’Meara, *et al*.^26^ and Roemer, *et al*.^19^ identified 634 and 567 genes as essential for growth, respectively, by using the GRACE library. Furthermore, Roemer, *et al*.^19^ estimated that *C. albicans* had around 1400 (21%) essential genes in its genome. These conflicting results may be explained by the use of different growth conditions by the studies and also by the response to activation of the Tet promoter which could still keep a basal level of gene expression allowing temporary growth. This phenomenon could also explain the phenotypes of the second group of conditional mutants identified. The conditional mutants for the genes *ARC40, ARC35*, *ORF19.2438*, and *SKP1* were not defective for planktonic growth while they produced statistically less biofilm as attested by lower metabolic activity or direct biofilm analysis. These genes are required for general cell processes which could affect any step of cell development, but intriguingly, the deletion of these genes did not affect free-floating cell growth in YPD over a 48 hour period though they are annotated as producing inviable nulls in *C. albicans*^20^. In the same group, the conditional repression of the delta(24)-sterol C- methyltransferase (*ERG6*) gene impacted biofilm metabolic activity and biomass with no detrimental effect on the growth of free-floating cells. The screening of the GRACE collection for filamentation demonstrated that the conditional repression of *ERG6* severely affected filamentation in different hyphal-inducing conditions, including hyphae growth in RPMI, and these filamentation defects could also impair biofilm development as demonstrated by the deletion of genes involved in filamentation^8,26,27^.

The repression of *ILS1* gene blocked yeast cell growth in planktonic condition, as expected as aminoacyl tRNA- synthetase genes are essential. The GRACE library has 23 heterozygous mutants for other amino acyl-tRNA synthetase genes. Twelve of them were identified as essential for growth and two conditional mutants for *ORF19.3341* and *DED81* were selected for the second screening; however, these conditional mutants were unable to grow in liquid media and also formed poor biofilms being grouped in the first set of conditional strains (Supp. 2 Figs 1, 2). In addition, the drug BAY 10-8888, a cyclic β-amino acid, which inhibits isoleucyl tRNA synthetase results in the inhibition of protein synthesis and cell growth in *C. albicans*^28^. However, cells with the *ILS1* gene shut off were capable of forming a unique mature biofilm structure. Our findings address the implication that *ILS1* gene is a conditional essential gene. To test this, the *ILS1* biofilm was investigated at the transcription level. It is possible the physical structure of the sessile community could prevent the repressive action of doxycycline of reaching the biofilm cells, but this appears not to be true. The concentration of doxycycline used in this study was able to inhibit 22 conditional mutants which were defective for both planktonic and biofilm growth demonstrating that the drug was able to reach the biofilm cells as much as it did for planktonic cells. Furthermore, *ILS1* was one of the most down-regulated genes in the *ILS1* biofilm compared with the wild-type biofilm, so the strain was clearly responsive to the doxycycline repression. It is not clear how *Candida* cells can overcome the absence of an essential gene in a complex mode of growth. Three putative aminoacyl tRNA- synthetase genes, *ORF19.3341, GUS1*, and *FRS2*, were de-repressed in the *ILS1* biofilm compared with the wild-type biofilm. Aminoacyl tRNA- synthetases bind their specific amino acids to their tRNA and are the only biological molecules able to read the genetic code; in addition, they can also have other functions, such as supporting RNA splicing, cell signaling, transcriptional and translational regulation^29^. In *C. albicans* the tRNA_CAG_^Ser^ is charged with both serine and leucine with preference for the amino acid serine. The amino acid leucine is misincorporated in 3 to 5% of the CUG codons under standard and mild stress conditions^30,31^. Perhaps the de-repressed aminoacyl tRNA- synthetase genes, *ORF19.3341, GUS1*, and *FRS2*, are potential candidates to cover *ILS*1 function. The repression of *ILS*1 also up-regulated genes annotated for translation, ribosome biogenesis, and RNA metabolic process, perhaps a response directed at maintaining a suitable level of protein synthesis in the face of *ILS1* perturbation.

The molecular basis of the *ILS1* biofilm in the mature stage appeared closely related to the wild-type biofilm as measured by the overall transcriptional profiles. However, there were some key differences. In order to thrive in the absence of *ILS1*, *Candida* biofilm cells differentially up-regulated some transcription factors, such as *CAS5, ACE2*, and *UME6*, which besides playing roles for biofilm formation are specifically required for cell wall organization, adhesion to plastic, and hyphae formation and dispersal control^32–36^. A set of specific GPI-anchored cell wall proteins were also up-regulated, including *EAP1* that was not differentially expressed in the wild-type biofilm compared with the WT planktonic cells, but was the second most up-regulated gene (9.27-fold higher) in the *ILS1*-shut off biofilm. This gene encodes a glycosylphosphatidylinositol-anchored, glucan-cross-linked cell wall protein, required for adhesion to plastic and for cell-cell interaction^37,38^. Overall the *ILS1* conditional biofilm was extremely sticky to the plastic, and it was difficult to detach the biofilm mass from surfaces. The adhesion-like protein *SMI1* and the exo-glucanase gene *XOG1* were also up-regulated. They are involved in manufacturing matrix β-1,3-glucan and the delivery and organization of mature biofilm matrix, respectively^39,40^. The gene that encodes an inhibitor of matrix production, *CSH1*, was up-regulated though its transcription regulator, *ZAP1*, was not induced in the *ILS1* biofilm but was induced in the wild-type biofilm^41^. The differential expression of these positive and negative extracellular matrix genes may have contributed to the balance of matrix production in the *ILS1* biofilm.

In biofilms, the repression of the molecular chaperone *HSP90* reduced the biofilm cell dispersion and the susceptibility to the antifungal azole without causing alteration in the biofilm architecture^42^. The depletion of *HSP90* function had no effect on the expressions of the transcription factors *BCR1, MIG1, TEC1, TUP1*, and *UPC2* during biofilm formation indicating that the *HSP90* biofilm interactors are still to be identified^43^. The *ILS1* biofilm up-regulated the expression of *HSP90* while the wild-type biofilm down-regulated it. This difference may have occurred due to the attempt of the conditional mutant to deal with the stress caused by the lack of isoleucyl-tRNA synthetase and its consequences.

Even though forming a mature biofilm structure, the *ILS1* – shut off biofilm demonstrated some notable weaknesses. The downregulation of genes involved in the processes related to morphology transition and the defective planktonic hyphal growth phenotype in RPMI-MOPS media and in serum as reported by O’Meara, *et al*.^26^ could be associated with the abnormal appearance of the cells in the *ILS1* biofilm. The down-regulation of cell cycle genes important for mitotic spindle formation and mitotic exit (Supp. 2 Fig. 4, Supp. 3) could also contribute to the defective phenotypes observed for the *ILS1* conditional mutant in both yeast and hyphal forms as described for the lack of mitotic regulators^44^. Moreover, the metabolic activity of the *ILS1* biofilm was considerably lower (*P* < 0.01) compared to the WT (−/+DOX) and heterozygous biofilms. The metabolic activity of the biofilm cells was evaluated by the conversion of XTT to a colored formazan which is performed by the mitochondrial succinoxidases, cytochrome P450 systems, and flavoprotein oxidases^45^. Although the overall expression of metabolic genes was not altered in the *ILS1* biofilm (Supp. 3), the gene *SDH2* that encodes a succinate dehydrogenase was significantly repressed at 1.15-fold (adjusted p-value < 0.0001) in the *ILS*1 biofilm. In addition, genes annotated for cellular homeostasis, such as iron metabolism genes *IRO1, CFL2, FTR1*, and *HAP43* were also down-regulated in the *ILS1* biofilm. Since 47% of enzymes are estimated to require metals for function as well as *SDH2*, the repression of genes required for metal metabolism might affect the biofilm cell metabolism^46^.

The biofilm screening of the GRACE strain collection revealed three distinct groups of genes that impacted on biofilm formation: a group of likely essential genes which were required for normal growth and in consequence affect biofilm formation (22 genes, Supp. 2 Figs 1, 2); putative essential genes or genes involved in general processes that when conditionally shut off still enabled growth, but formed less biofilm (*ARC40, ARC35, ORF19.2438, SKP1, ERG6*, and *ADE5,7*), and intriguingly, a conditionally essential genes, *ILS1*, which when supressed was unable to grow in normal conditions but still formed a biofilm comparable to those of WT cells and relying on the classical biofilm “building block” genes but differentially regulating a unique set of genes to bypass the need of an aminoacyl-tRNA synthetase.

## Methods

### Strains and media

The clinical isolate SC5314 was used as the wild-type (WT) strain throughout the experiments^47^. The GRACE collection containing 2358 strains (*orfX::his3::hisG/his3::hisG leu2::tetRGAL4AD-URA3/LEU2*) and the wild- type strains were routinely cultured in YPD medium (1% yeast extract, 2% bacto-peptone, 2% D-glucose, [2% agar])^19^. The biofilms were grown in RPMI medium (Hyclone, Logan, USA) buffered with MOPS (Bioshop, Burlington, Canada) (0.165 M, pH 7.0) supplemented or not with 20 mg l^−1^ doxycycline (DOX). All the media above were supplemented with 50 mg l^−1^ uridine.

### Determining gene essentiality

The GRACE strains were pre-inoculated in 100 µL of liquid YPD and incubated at 30 °C overnight. The cultures were diluted at approximately 1.3 × 10^3^ cells and transferred to plates with either YPD agar or YPD agar containing 100 µg mL^−1^ tetracycline. The addition of tetracycline to the media induces the tet-repressed state of the library; this shuts off each specific gene thus mimicking the effect of a homozygous deletion^19,48^. The plates were then incubated at 30 °C for 48 h. Strains were classified as essential or non-essential based on their growth.

### Biofilm growth

For the first screening with the GRACE library, the strains were grown overnight in YPD at 30 °C in non-treated surface 96-well plates. The cells were transferred to fresh media and the plates incubated at 30 °C for 4 h with or without DOX. To grow biofilms, 8 µL of the cultures (~10^4^–10^5^ cells mL^−1^) were transferred to RPMI-MOPS medium with or without doxycycline in 96-well plates. The plates were incubated at 37 °C/48 h under static conditions. The biofilm growth was washed with phosphate buffered saline (PBS) and, then, measured at OD_600_ using a microtiter plate reader (Tecan Infinite M200 Pro). The liquid handling was performed by a liquid-handling robot (Biomek FX). The selected strains were screened again for further biofilm features as described with some modifications^25^. The selected GRACE strains were cultured in YPD liquid at 30 °C for 24 h with 220 rpm shaking. Then the cells were transferred to fresh YPD medium containing or not DOX and incubated at 30 °C overnight with shaking. The cells were washed with PBS and resuspended in RPMI-MOPS medium with or without DOX. The concentration of cells was adjusted to OD_600_ = 0.5. Two-hundred microliters of the inoculum was transferred to a well of 96-well plate for initial incubation at 37 °C for 1.5 h in static conditions to allow cell adherence. Next, the non-adherent cells were washed off with PBS and fresh RPMI-MOPS medium with or without doxycycline was added. The plates were incubated for 48 h at 37 °C with 75 rpm shaking.

### Biofilm analysis

The biofilms were washed once with PBS prior to measurement of OD_600_, metabolic activity and biomass. The metabolic activity of the biofilms was monitored by the 2,3-bis (2-methoxy-4-nitro-5-sulfophenyl)-5-[(phenylamino) carbonyl]-2H-tetrazolium hydroxide (XTT)- colorimetric assay as described^49^. Each well received 158 µL of PBS, 40 µL of 1 mg mL^−1^ XTT solution (Sigma), and 2 µL of fresh 0.4 mM of menadione solution (Sigma). The plates were incubated at 37 °C for 3 h with shaking in the dark. Following, 100 µL of the supernatant was transferred to a new well and the colorimetric change was read at 492 nm using a microtiter plate reader. The biomass accumulation was examined by the crystal violet (CV) assay^50^. Briefly, the biofilms were air dried for 45 min and, then stained with 0.4% aqueous CV solution for 45 min at room temperature (RT). Next, the biofilms were washed twice with sterile water, and then de-stained with 95% ethanol for 45 min at RT. The de-stained solution was measured at OD_495_. To compare with studies that used OD_595_, the absolute numbers may be multiplied by the factor 12.

To determine the biofilm dry weight^51^, the biofilms were grown on non-tissue treated surface 6-well plate as described above and the media and PBS volumes were adjusted to 4 mL. After washing the biofilms with PBS, 2 mL of PBS was added to each well and the biofilms were disrupted by pipetting up and down. The contents were filtered over pre-weighed 0.8 µm nitrocellulose filters (Millipore AAWG02500). A well containing only media was included as a control. The biofilm-containing filters were dried out overnight and weighed the day after. The weight of the filter was subtracted from the weight of the biofilm-containing filter to get the biofilm dry weight.

Disaggregation assays were performed with 24 h-biofilms (biofilm growth method)^17,39^. The biofilms were washed with PBS, and then treated with 100 µL fresh media plus 100 µL of Zymolyase (20,000 units/g, Bioshop) in 0.9% NaCl at concentrations of 0.625 and 1.25 units mL^−1^ or non-treated (fresh media) for additional 24 h at 37 °C in static condition. The biofilms were washed with PBS and stained with crystal violet for quantification.

Scanning electron microscope (SEM) was used to assess the biofilm architecture. The biofilms were grown on 1.5 by 1.5 cm silicon squares (1/16” th white (FDA) silicone 6” wide X 6” long, Canada Rubber Group Inc.), which were pre-treated with bovine serum. The treated silicon squares were washed with PBS and inoculated with the *Candida* cells. The biofilm method used was described above^25^ and the media and PBS volumes were adjusted to 2 mL. After 48 h incubation, the biofilm-containing silicon squares were washed with PBS and fixed in 2.5% glutaraldehyde (Sigma-Aldrich) for 1 h, washed again with PBS, and then placed in 1% osmium tetroxide for 30 min. Subsequently the biofilms were dehydrated by a series of 10 min ethanol washes (30, 50, 70, 85, 95, and 100%) and dried out overnight. The samples were mounted on aluminium stubs, sputter coated with 60% gold and 40% palladium, and imaged with a Hitachi S-3500 scanning electron microscope.

### Growth curves

The strains, pre-cultured in YPD liquid and then in YPD liquid −/+ DOX at 30 °C for overnight with shaking, were washed with PBS and adjusted to OD_600_ = 0.05 in YPD −/+ DOX. The inocula were added to a 96-well plate to measure the growth at OD_595_ at 30 °C/37 °C for 48 h in a microtiter plate reader. The growth measurements were performed in triplicate on three different occasions (Tecan).

### Cell microscopy

To examine cell morphology, the strains were first cultured in YPD at 30 °C/24 h with 220 rpm shaking. Then, the cells were transferred to YPD to grow planktonic yeast cells at 30 °C or RPMI-MOPS or Spider medium (1% nutrient broth, 1% mannitol, 0.4% K_2_HPO_4_, pH 7.2) to induce planktonic hyphal growth at 37 °C −/+ DOX and incubated overnight with 220 rpm shaking. The cells were washed and resuspended in PBS, and imaged using LEICA_DM6000 microscope at 1000x and 100x magnification, respectively. The DIC images were analyzed by FIJI-win64 and the yeast cell volume determined as described by Malavia, *et al*.^52^.

### RNA-seq

The biofilms were grown on 6-well plates with non-treated surfaces for 48 h as described above. One entire 6-well plate was used per sample. Next, the supernatants were removed and 2 mL of PBS was added to each well. The biofilms were disrupted by gently pipetting up and down along the bottoms of the well. The biofilm slurries of the same sample were combined in 15 mL tubes and centrifuged. The wild-type culture in YPD incubated at 30 °C/overnight was used as the planktonic growth condition. The total RNA was extracted using the QIAGEN RNA extraction kit protocol with the exception that the cells were disrupted completely with bead beater shaking for 10 times for 20 seconds with 1 min cooling on ice between treatments. Samples were tested for quality control using a Bioanalyzer, and submitted to the Genome Quebec Innovation Centre for sequencing using an Illumina miSeq sequencing platform. RNA-Seq data was processed as described^53^. Raw and processed data have been deposited in NCBI’s Gene Expression Omnibus and are accessible through GEO Series accession number GSE127796 (https://www.ncbi.nlm.nih.gov/geo/query/acc.cgi?acc=GSE127796)^54^. We imported gene abundances into R and conducted differential analysis of gene count data using the DESeq. 2 R package^55^. We normalized gene count data using the vst variance stabilizing transformation implemented in DESeq. 2 dealing with library size and within-group variability of low counts^55^. Unsupervised principal component analysis (PCA) was performed on normalized gene expression and each profile was plotted based on the scores of the first two principal components. Mean expression of every gene was calculated within each group (WT planktonic cells, WT biofilm, *ILS1* biofilm) and depicted in a heatmap where rows and columns were ordered by average linkage hierarchical clustering using correlation as a distance measure.

### Analysis of results

For the first biofilm screening, the OD_600_ values obtained for the biofilms formed by the heterozygous mutants were subtracted from the values obtained for the conditional mutants. The conditional mutants that showed poor growth in the gene essentiality test were ruled out of the screen. In addition, the results were scanned by eye to select the best hints. The results obtained in the second screening were analyzed by analysis of variance (ANOVA) and the Tukey test or Student’s *t* test. GraphPad Prism version 6.00 was used for the analysis of results.

## Supplementary information

Dataset 1

Dataset 2

Dataset 3
