# Supplementary material for: Screening of Candida albicans GRACE library revealed a unique pattern of biofilm formation under repression of the essential gene ILS1
Source: Sci Rep. 2019 Jun 24;9:9187. doi: 10.1038/s41598-019-45624-y (PMC6591175; doi:10.1038/s41598-019-45624-y)
Supplement: Supplementary file 2 — Dataset 2 [file 41598_2019_45624_MOESM2_ESM.docx]

**Screening of *Candida albicans* GRACE library revealed a unique pattern of biofilm formation under repression of the essential gene *ILS1***

Anna Carolina Borges Pereira Costa^1^, Raha Parvizi Omran^1^, Tuana Oliveira Correia-Mesquita^1,2^, Vanessa Dumeaux^3^, Malcolm Whiteway^1*^

^1^Department of Biology, Concordia University, Montreal, Canada.

^2^Department of Biology, McGill University, Montreal, Canada.

^3^PERFORM Centre, Concordia University, Montreal, Canada.

Anna Carolina Borges Pereira Costa: Department of Biology, Concordia University. 7141 Sherbrooke St W., Montreal, Quebec, Canada, H4B 1R6. E-mail: [carol_biolog@yahoo.com.br](mailto:carol_biolog@yahoo.com.br)

Raha Parvizi Omran: Department of Biology, Concordia University. 7141 Sherbrooke St W., Montreal, Quebec, Canada, H4B 1R6. E-mail: [raha.parvizi.o@gmail.com](mailto:raha.parvizi.o@gmail.com)

Tuana Oliveira Correia-Mesquita: Department of Biology, McGill University. 1205 Docteur Penfield Street, Montreal, Quebec, Canada, H3A 1B1. E-mail: tuana.oliveira@gmail.com

Vanessa Dumeaux: PERFORM Centre, Concordia University. 7200 Sherbrooke St. W.

Montreal, Quebec, Canada, H4B 1R6. E-mail: vanessa.dumeaux@concordia.ca

*Corresponding author:

Malcolm Whiteway: Department of Biology, Concordia University. 7141 Sherbrooke St W., Montreal, Quebec, Canada. H4B 1R6. Phone: +1 (514) 848-2424 ext. 3751. E-mail: malcolm.whiteway@concordia.ca

**Supplemental material 2**

**Figure 1. Twenty-two conditional mutants were defective for biofilm formation.** Biofilm growth (OD_600_) measurement (A), metabolic activity determination (XTT- assay) (B), and biomass accumulation (Crystal violet straining assay) (C). The biofilms were grown in RPMI-MOPS at 37^o^C for 48 h -/+DOX. The experiments were performed in triplicate on three different occasions. *P < 0.05, **P < 0.01, ***P < 0.001, ****P < 0.0001, *t* test.


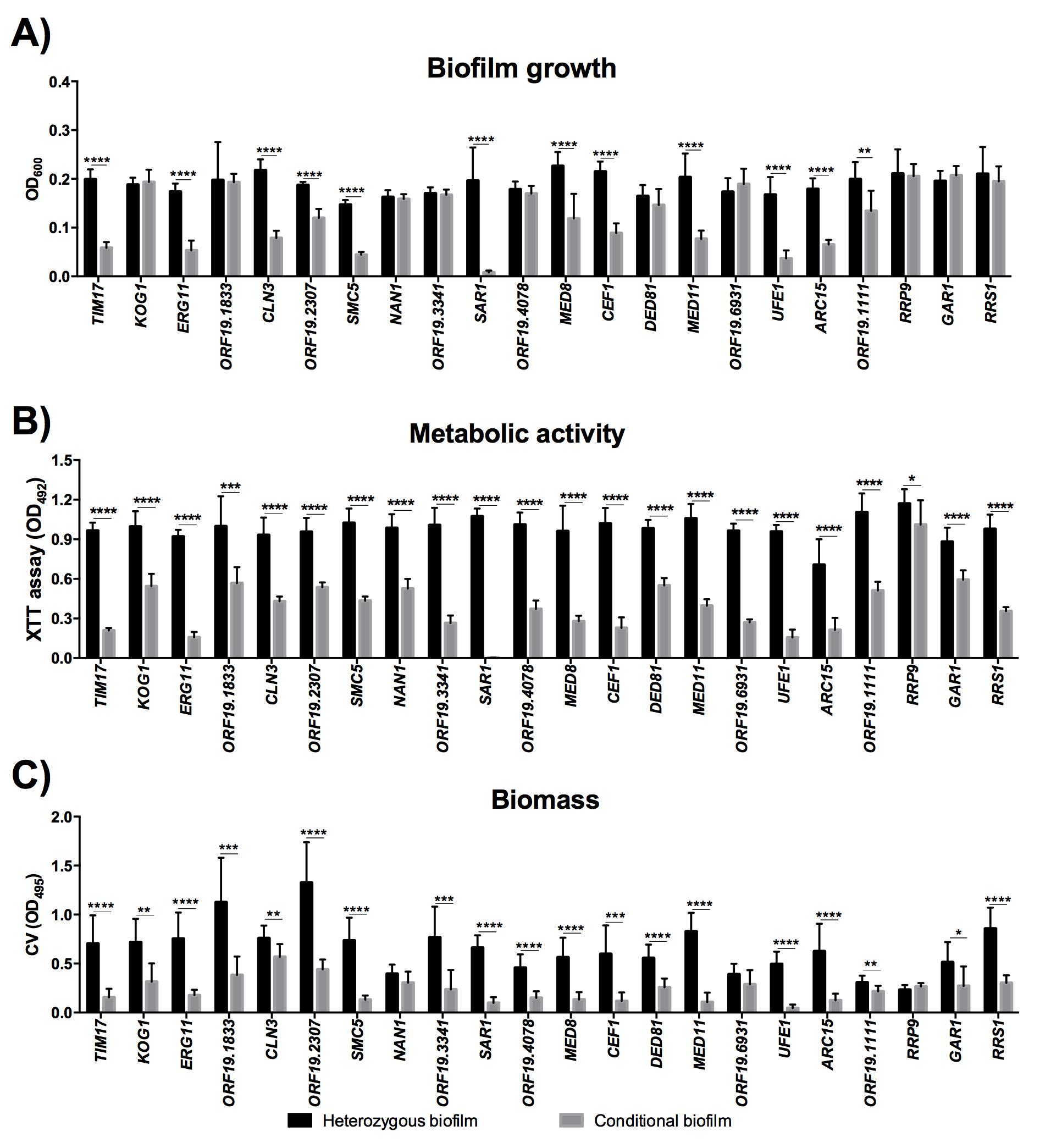


**Figure 2. Twenty-two conditional mutants were deficient for normal growth *in vitro.*** Planktonic growth curve for the strains grown in YPD -/+DOX at 30^o^C for 48 h. The experiments were performed in triplicate on three different occasions.


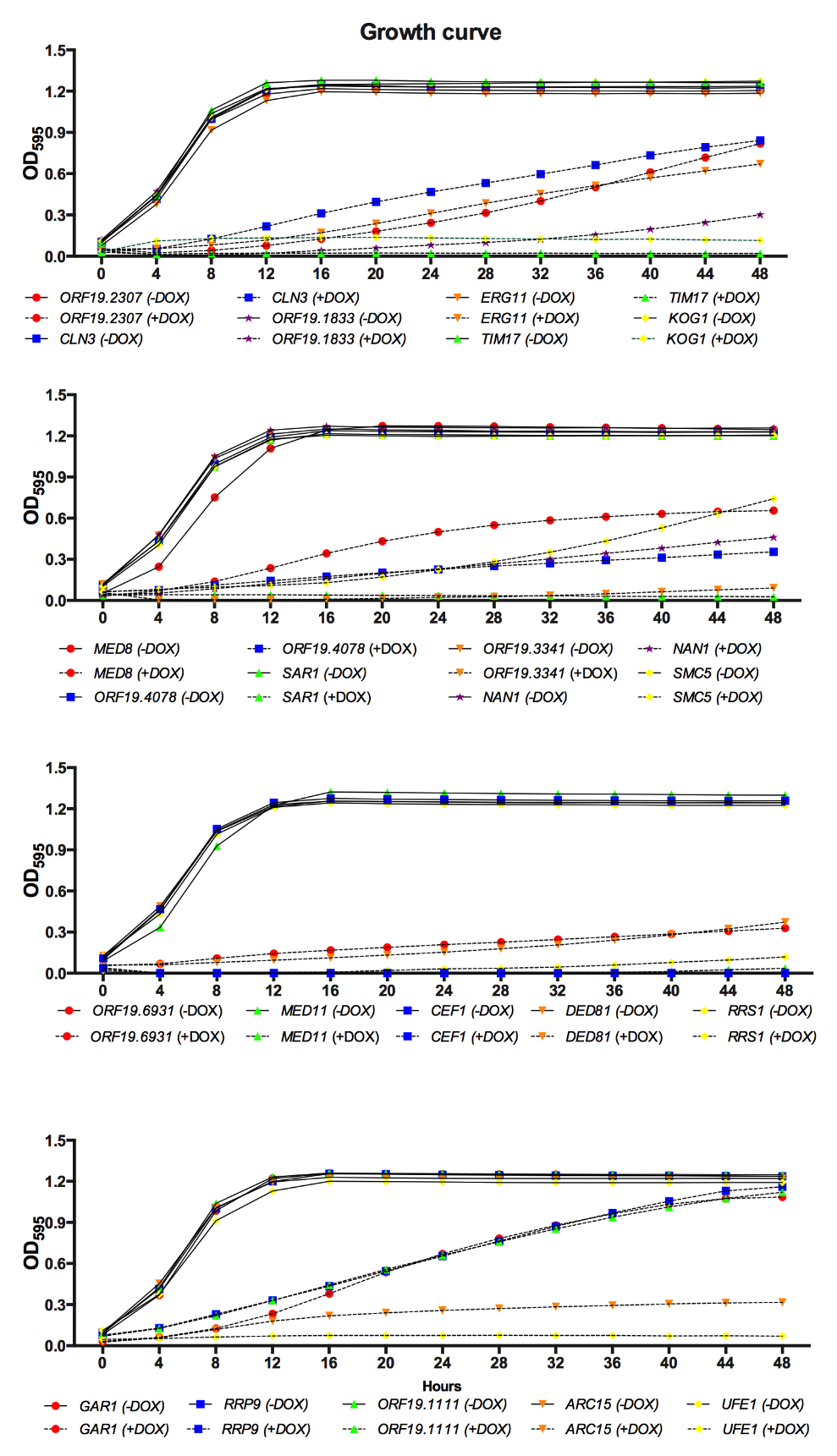


**Table 1. Summary of gene descriptions and biofilm findings.**

| Gene | CGD^a^ | SGD^b^ | Biofilm feature^c^ |
| --- | --- | --- | --- |
| *ARC40 (ORF19.3873)* | ORF verified. Protein similar to *Saccharomyces cerevisiae* Arc40; involved in actin filament organization in *S. cerevisiae*; transposon mutation affects filamentous growth; rat catheter and Spider biofilm repressed. Inviable null. | ARC40. ORF verified. Subunit of the ARP2/3 complex; ARP2/3 is required for the motility and integrity of cortical actin patches. Viable/Inviable null. | Reduced biomass and statistically reduced biofilm metabolic activity (*P* < 0.05). |
| *ARC35 (ORF19.2437)* | ORF verified. Putative ARP2/3 complex subunit; shows colony morphology-related gene regulation by Ssn6p; mutation confers hypersensitivity to cytochalasin D. Inviable null. | ARC35. ORF verified. Subunit of the ARP2/3 complex; ARP2/3 is required for the motility and integrity of cortical actin patches; required for cortical localization of calmodulin. Inviable null. | Reduced biofilm growth (OD_600_) (*P* < 0.001) and metabolic activity (*P* < 0.01), but comparable biomass. |
| *ERG6 (ORF19.1631)* | ORF verified. Delta(24)-sterol C-methyltransferase, converts zymosterol to fecosterol, ergosterol biosynthesis; mutation confers nystatin resistance; Hap43, GlcNAc-, fluconazole-induced; upregulated in azole-resistant strain; Spider biofilm repressed. Viable null. | ERG6. ORF verified. Delta(24)-sterol C-methyltransferase; converts zymosterol to fecosterol in the ergosterol biosynthetic pathway by methylating position C-24; localized to lipid particles, the plasma membrane-associated endoplasmic reticulum, and the mitochondrial outer membrane. Viable null. | Statistically reduced metabolic activity (*P* < 0.05) and biomass accumulation (*P* < 0.05). |
| *ORF19.2438* | ORF uncharacterized. Ortholog(s) have mitochondrion, ribosome localization. Inviable null. | MRPS12. ORF verified. Mitochondrial protein; may interact with ribosomes based on co-purification experiments; similar to *E. coli* and human mitochondrial S12 ribosomal proteins. Viable null. | Reduced biomass and statistically reduced biofilm metabolic activity (*P* < 0.01). |
| *SKP1 (ORF19.4427)* | ORF uncharacterized. Putative subunit D of kinetochore protein complex CBF3; regulated by Gcn4p; repressed in response to amino acid starvation (3-aminotriazole treatment). Inviable null. | SKP1. ORF verified. Evolutionarily conserved kinetochore protein; part of multiple protein complexes, including the SCF ubiquitin ligase complex, the CBF3 complex that binds centromeric DNA, and the RAVE complex that regulates assembly of the V-ATPase; protein abundance increases in response to DNA replication stress. Inviable mutant. | Statistically reduced biofilm growth (*P* < 0.05) and metabolic activity (*P* < 0.001). |
| *ADE5,7 (ORF19.5061)* | ORF verified. Phosphoribosylamine-glycine ligase and phosphoribosylformylglycinamidine cyclo-ligase; interacts with Vps34p; required for hyphal growth and virulence; flucytosine induced; not induced in GCN response, in contrast to S. cerevisiae ortholog . Viable null. | ADE5,7. ORF verified. Enzyme of the 'de novo' purine nucleotide biosynthetic pathway; contains aminoimidazole ribotide synthetase and glycinamide ribotide synthetase activities. Viable null. | Statistically reduced biofilm growth (*P* < 0.01), metabolic activity (*P* < 0.01) and biomass accumulation (*P* < 0.05). |

^a^CGD (*Candida* genome database) (http://www.candidagenome.org/)^1^

^b^SGD (*Saccharomyces* genome database) (http://www.yeastgenome.org)^2^

^c^Description of the results based on statistical difference.

**Figure 3. Planktonic hyphal growth in Spider medium.** The WT and the *ILS1* GRACE strains were grown in Spider medium (-/+DOX) at 37^o^C/ overnight/ 220 rpm and imaged to determine the cell morphology. DIC representative images for each strain, scale bars represent 100 µm.

**
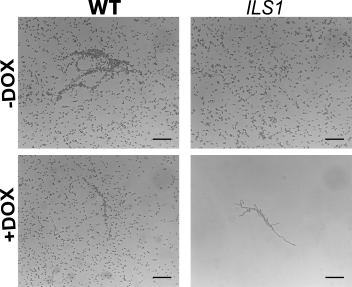
**

**Figure 4. Process categorisation of the up-regulated and down-regulated genes in *ILS1* biofilm compared with the WT biofilm.** The up-regulated (>1.5 fold-change, adjusted p-value < 0.01) and down-regulated (<-1.5 fold-change, adjusted p-value < 0.01) genes in *ILS1* conditional biofilm were categorised using the Process Ontology in the CGD Gene Ontology Slim Mapper.

**

**

**Reference**

1. Inglis, D. O. *et al*. The *Candida* genome database incorporates multiple *Candida* species: multispecies search and analysis tools with curated gene and protein information for *Candida albicans* and *Candida glabrata*. *Nucleic Acids Res.* **40**, D667-74 (2012).

2. Cherry, J. M. *et al*. Saccharomyces Genome Database: the genomics resource of budding yeast. *Nucleic Acids Res.* **40**, D700-5 (2012).
